# Efficacy of aldose reductase inhibitors is affected by oxidative stress induced under X-ray irradiation

**DOI:** 10.1038/s41598-019-39722-0

**Published:** 2019-02-28

**Authors:** Albert Castellví, Isidro Crespo, Eva Crosas, Ana Cámara-Artigas, José A. Gavira, Miguel A. G. Aranda, Xavier Parés, Jaume Farrés, Judith Juanhuix

**Affiliations:** 1 Alba Synchrotron, carrer de la Llum 2-26, 08290 Cerdanyola del Vallès, Barcelona, Catalonia Spain; 2 Department of Chemistry and Physics, Universidad de Almería, 04120 Almería, Spain; 3 Laboratorio de Estudios Cristalográficos, Instituto Andaluz de Ciencias de la Tierra, CSIC-Universidad de Granada, Avenida de las Palmeras 4, 18100 Armilla, Granada Spain; 4 Department of Biochemistry and Molecular Biology, Faculty of Biosciences, Universitat Autònoma de Barcelona, 08193 Bellaterra, Barcelona, Catalonia Spain; 5 Present Address: Deutsches Elektronen-Synchrotron DESY, Photon Science, Notkestrasse 85, 22607 Hamburg, Germany; 6 Present Address: Department of Biochemistry and Molecular Biology, Faculty of Biosciences Universitat Autònoma de Barcelona, 08193 Bellaterra Barcelona, Catalonia, Spain

## Abstract

Human aldose reductase (hAR, AKR1B1) has been explored as drug target since the 1980s for its implication in diabetic complications. An activated form of hAR was found in cells from diabetic patients, showing a reduced sensitivity to inhibitors in clinical trials, which may prevent its pharmacological use. Here we report the conversion of native hAR to its activated form by X-ray irradiation simulating oxidative stress conditions. Upon irradiation, the enzyme activity increases moderately and the potency of several hAR inhibitors decay before global protein radiation damage appears. The catalytic behavior of activated hAR is also reproduced as the K_M_ increases dramatically while the *k*_*cat*_ is not much affected. Consistently, the catalytic tetrad is not showing any modification. The only catalytically-relevant structural difference observed is the conversion of residue Cys298 to serine and alanine. A mechanism involving electron capture is suggested for the hAR activation. We propose that hAR inhibitors should not be designed against the native protein but against the activated form as obtained from X-ray irradiation. Furthermore, since the reactive species produced under irradiation conditions are the same as those produced under oxidative stress, the described irradiation method can be applied to other relevant proteins under oxidative stress environments.

## Introduction

The increase in the flux of the polyol pathway is the main mechanism by which hyperglycemia leads to diabetic complications, such as microvascular and macrovascular damage. This pathway is followed by only 3% of the cytosolic glucose in physiological conditions but increases to more than 30% under hyperglycemic conditions^1,2^. The key step in this pathway is the reduction of glucose to sorbitol catalyzed by aldose reductase (hAR, AKR1B1), a member of the NAD(P)H-dependent aldo-keto reductase superfamily^1–6^. One of the first cellular deleterious effects is NADPH depletion, reducing its availability for the regeneration of the powerful antioxidant glutathione (GSH) and weakening intracellular antioxidant defenses^2,5,7,8^. Moreover, in the second step of the polyol pathway, sorbitol is metabolized by sorbitol dehydrogenase to fructose at the expense of producing additional NADH, which potentially increases the reactive oxygen species (ROS) via NADH oxidase and electron leakage in the electron transport chain^8,9^. The inhibition of hAR would therefore block the polyol pathway, counteracting significantly the unbalanced conditions caused by intracellular oxidative stress typical of diabetes.

Aldose reductase inhibitors (ARIs) have been indeed the focus of research for over 40 years since the discovery of the involvement of hAR in diabetic processes^2^. Unfortunately, the attempts to find an effective drug targeting hAR under diabetic conditions have been unsuccessful so far. Although several inhibitors have been reported to effectively block the hAR activity *in vitro* using purified protein^7^, their potency is severely reduced when tested in clinical trials. Early on, this effect was hypothesized to be related to the generation, in significant amounts, of an activated form of hAR under oxidative stress conditions, which differs from the native form in its kinetic constants and a marked reduction to ARIs sensitivity^10–12^. Remarkably, it has been shown that hAR can be activated also *in vitro* by mutagenesis of Cys298 residue (C298S^13,14^, C298A^14,15^), which has been identified as the main target for various oxidative modifications^16–19^, or by the incubation of the enzyme with chemical compounds^20^. The study of ARIs therefore requires the use of this hAR form present under intracellular oxidative stress conditions found in diabetes, which differs from the native form expressed in physiological conditions or prepared by recombinant techniques.

The oxidative stress causing metabolic abnormalities in cells exposed to hyperglycemia is mainly produced by the accumulation of intracellular free electrons and reactive oxygen species (ROS), notably O_2_^●−^, H_2_O_2_ and OH^●^, among others^21^. Cellular oxidative stress can not be reproduced *in vitro* due to the complexity and diversity of factors influencing this condition. In consequence, *in vitro* studies under oxidative stress conditions utilize indirect methods to produce the same reactive species as those produced *in vivo*, such as the addition of chemical compounds. Another common method, which bypasses the potential bias in the result interpretation that the use of a particular chemical compound might introduce, is to employ electrons or ionizing radiation to create radical species in protein solutions^22–28^. As documented extensively in the literature, the radical species induced by irradiation are the same than those present under oxidative stress conditions^21,29^. The use of X-rays of a wavelength of ~1 Å (12.4 keV) is particularly convenient as the energy is deposited evenly over a thickness in the millimiter range or less in protein solutions or crystals. Here we employ synchrotron X-ray irradiation at that wavelength to report a modification of hAR which reproduces the behavior of the activated form generated under diabetic oxidative stress conditions. This modification has been characterized by enzymatic assays after exposition of hAR in solution under different irradiation doses, by monitoring the progressive structural changes of hAR crystals as irradiation dose increases and by characterizing the structural changes in solution using mass spectrometry. The activity of the *in-vitro* potent ARIs Zenarestat^30^, JF0048^31^, Epalrestat^32^ and Tolrestat^33^ was also checked at different irradiation doses. A mechanism for the activation of hAR under oxidative stress conditions is proposed and some suggestions for the structure-based drug design targeting hAR are provided.

## Results

### X-ray irradiated hAR behaves as the activated form in enzymatic assays

**Figure 1:**
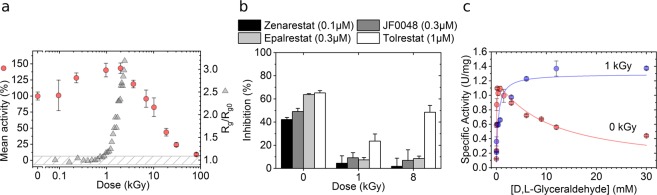
Activation of hAR with irradiation. (**a**) Evolution of hAR normalized mean activity (red circles) and radius of gyration, Rg (gray triangles), as a function of dose. At 6 mM D,L-glyceraldehyde substrate concentration, enzymatic activity increases with irradiation dose until hAR undergoes major structural changes due to radiation damage. (**b**) Inhibitory potency of Zenarestat (black bars), JF0048 (gray bars), Epalrestat (light gray bars) and Tolrestat (white bars) at 0 kGy, 1 kGy and 8 kGy doses. Tested concentrations close to the IC50 value were used for each compound. The non-normalized specific activities of the inhibitors are found in Supp. (**c**) Effect of irradiation on hAR kinetics. Native enzyme without irradiation loses 50% of its activity due to excess-substrate inhibition at 6 mM d, l-glyceraldehyde (red circles). Irradiation with a dose of 2 kGy increases the value of the Michaelis constant K_M_ towards substrate by near one order of magnitude (7 times) while the inhibition disappears (blue circles).

Enzymatic assays reveal that the activity of hAR at a concentration of 2.3 mg/mL increases moderately by ~50% when it receives a dose between 1 and 2 kGy, as compared to the non-irradiated protein, without significant general structural changes as shown by Small Angle X-ray Scattering (SAXS) measurements (Fig. 1a). The radius of gyration (R_g_) of hAR in solution remains constant within 0.1 nm variation, which is a well-stablished criterion to assess the preservation of the global protein structure under irradiation^34,35^, until the protein receives a dose of ~1 kGy. Above 2 kGy, the relative activity of hAR decays concomitantly with the dramatic increase of the radius of gyration as a consequence of radiation damage^36^. The inhibitory effect of *in-vitro* potent ARIs Zenarestat, JF0048, Epalrestat and Tolrestat was checked for non-irradiated hAR, for hAR irradiated to the point where the highest activity is reached without significant protein unfolding or aggregation (1 kGy) and for over-irradiated, damaged hAR (8 kGy) (Fig. 1b). While inhibiting native non-irradiated hAR, the studied compounds were significantly less effective in irradiated hAR. The kinetic analysis of non-irradiated and irradiated hAR at 1 kGy (Fig. 1c) shows that the catalytic constant k_cat_ value remains unaltered (*k*_cat_^0kGy^ = 45.0 ± 3.0 min^−1^ *vs* *k*_cat_^1kGy^ = 46.4 ± 3.5 min^−1^) while the Michaelis constant K_M_ value exhibits a 7-fold increase under irradiated conditions (K_M_^0kGy^ = 55.3 ± 13.1 µM *vs* K_M_^1kGy^ = 382.8 ± 141.6 µM). In agreement with previous reports^10,11,20,37^, non-irradiated hAR is inhibited by over-saturating concentrations of the substrate d,l-glyceraldehyde. Conversely, irradiated hAR is not inhibited by its substrate at any of the working substrate concentrations, as previously reported for the activated hAR^10,11,20,37^. This behavior is consistent with the higher activity of the irradiated protein with respect to native non-irradiated protein at 6 mM d,l-glyceraldehyde, while maintaining a similar *k*_*cat*_ value. In conclusion, the 1-kGy irradiated form of hAR solution at 2.3 mg/mL and the activated form of hAR found under hyperglycemic and activation conditions^12,38^ show the same behavior in the enzymatic analyses of the control hAR and the hAR in the presence of the ARIs. X-ray irradiation is thus able to reproduce the functionality of hAR under oxidative stress conditions.

### The only change in the crystallographic structure with accumulated dose is the modification of Cys298

The effects of the X-ray irradiation on the hAR structure were monitored by macromolecular crystallography (MX). Twenty datasets (D1-D20) were collected at 100 K from one single crystal of hAR co-crystallized with NADPH by a large homogeneous X-ray beam profile at the XALOC synchrotron beamline^39^. The absorbed dose per each data set in the exposed region was 0.03 MGy. The crystal was irradiated with an absorbed dose of 0.15 MGy between consecutive datasets, so that the total dose received by the crystal was 3.45 MGy. Data collection statistics for all datasets and refinement statistics for D1 and D20 datasets are shown in Sup. Table 1. Remarkably, the resolution of the datasets experiences a constant, modest degradation from 0.92 Å in dataset D1 to 1.17 Å in dataset D20. We incidentally note that the doses absorbed by the cryocooled crystals are not to be compared with the absorbed doses by the protein solution at room temperature, as the effects of the radiation damage strongly depend upon the protein concentration^40^ and the temperature^36,41^. Rather than comparing the actual dose, a coarse correspondence on the radiation damage effects can be established between experiments employing cryocooled protein crystals and those using protein solution at room temperature cas the global structure of the protein is preserved in MX and SAXS experiments, respectively.

**Figure 2:**
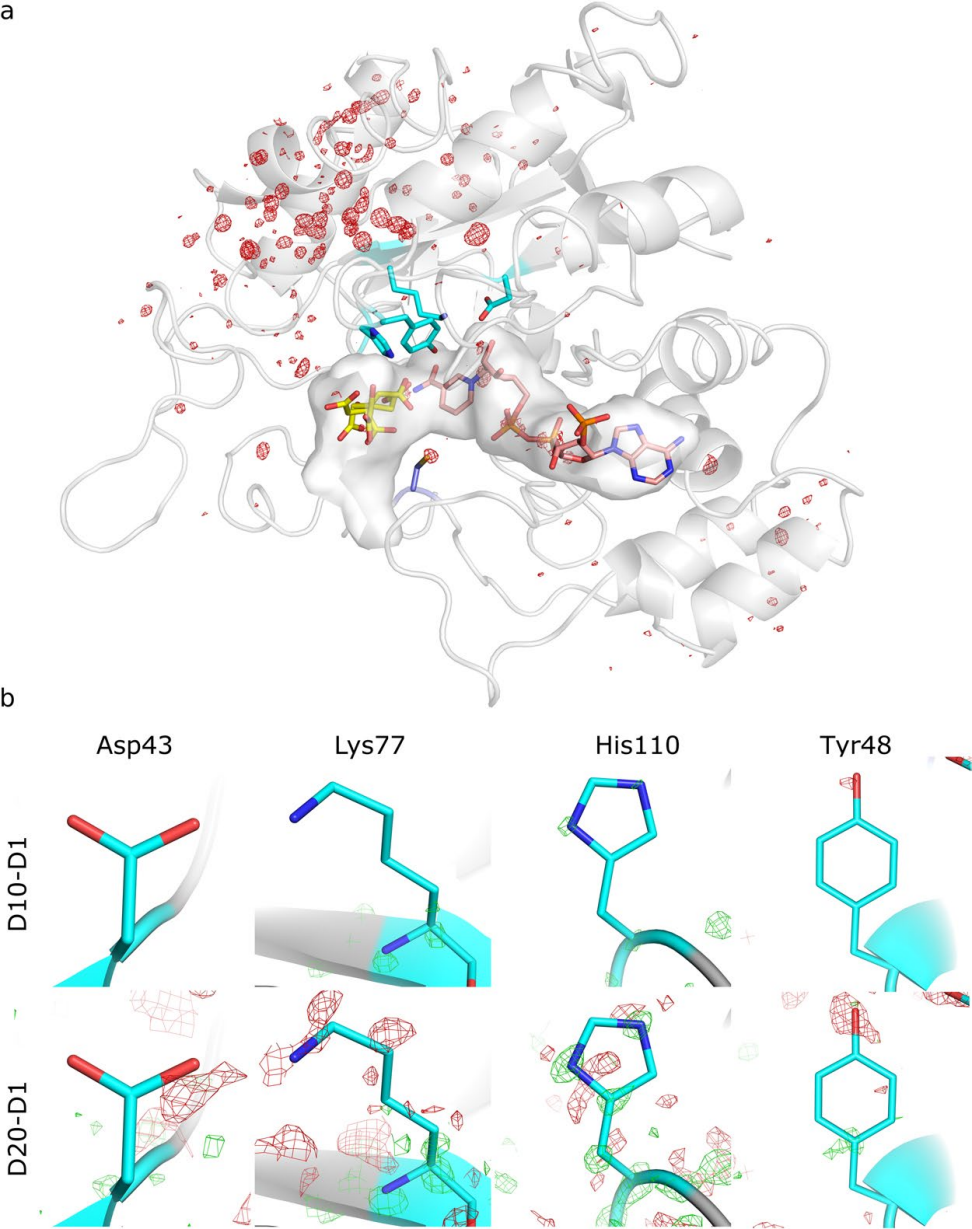
Absence of specific radiation damage in the binding pocket and in the catalytic tetrad. (**a**) Difference Fourier map (F_obs,20_ - F_obs,1_, α_calc,1_) of the most irradiated dataset, D20. The NADPH (pink) and a citrate molecule (yellow) are bound to the binding pocket. The residues of the catalytic tetrad are shown in cyan and Cys298 in navy blue. No signs of specific radiation damage are observed in the vicinity of the binding-pocket surface, except for the Cys298 residue. The map was contoured at 0.56 e/Å^3^ (σ = ±5). (**b**) Detail of the difference Fourier maps (F_obs,10_ - F_obs,1_, α_calc,1_) (top row) and (F_obs,20_ - F_obs,1_, α_calc,1_) (bottom row) on the catalytic tetrad (Asp43, Lys77, His110 and Tyr48) at 1.2 Å resolution, calculated using the amplitudes of datasets D10 or D20 and the initial data set D1 and the phases α_calc,1_ of D1. The maps do not reveal signs of specific radiation damage in the residues of the catalytic tetrad. The D10-D1 and D20-D1 difference Fourier maps of the catalytic tetrad were contoured at 0.33 e/Å^3^ (σ = ±4.97 and σ = ±2.97, respectively).

**Figure 3:**
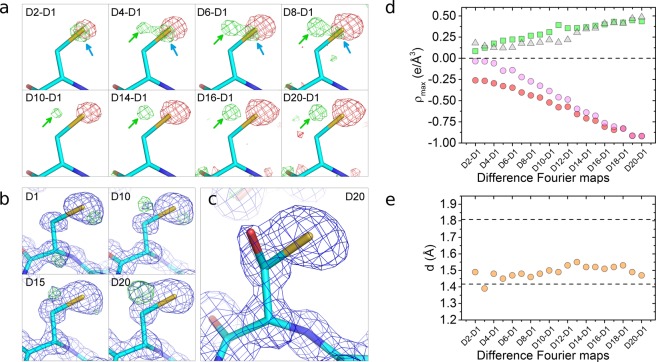
Specific changes in Cys298 as a function of dose. (**a**) Difference Fourier maps (F_obs,n_ - F_obs,1_, α_calc,1_) on Cys298 residue at 1.2 Å resolution, calculated between each dataset n and the initial dataset using the phases of the fresh, not previously irradiated dataset, α_calc,1_. The difference Fourier maps are contoured at two different electron density sigma levels (0.145 e/Å^3^ for the first row, 0.267 e/Å^3^ for the second row) for clarity. The maps have sigma levels ranging from σ = ±9.00 (D2-D1 map) to σ = ±2.53 (D8-D1 map) for the first row, and from σ = ±3.99 (D10-D1 map) to σ = ±2.41 (D20-D1 map) for the second row. Electron densities colored in green and red represent positive and negative sigma levels, respectively. Red blobs are indicative of the loss of electron density due to movements and desulfuration of Cys298; green blobs pointed by blue arrows show the increase of electron density due to displacements of Cys298 and green blobs pointed by green arrows reveal the appearance of a new atom in the vicinity of Cys298. (**b**) Electron density maps at 0.92–1.17 Å resolution on the Cys298 residue as a function of dose. In blue, 3Fo-2Fc electron density maps at σ = 1.00; in green, Fo-Fc electron density maps at σ = 2.50. (**c**) The Cys298/Ser298 amino acid is well fit in the 2Fo-Fc electron density map at σ = 1 for the D20 dataset. The Fo-Fc map at σ = ±2.50 does not show any electronic density. (**d**) Occupancy-related electron density maxima versus dataset. Red circles: occupancy-related electron density of Sγ atom decreases as Cys298 is desulfurated and the Sγ atom moves; green squares: occupancy-related electron density of Oγ atom increases as the Ser298 is formed; pink circles: loss of the electron density of the Sγ atom due to the desulfuration, calculated as the difference between the two previous electron densities; gray triangles: difference between the electron densities related to the desulfuration of Cys298 (pink circles) and the formation of the Ser298 (green squares). (**e**) Distances measured in the difference Fourier maps from the Cys298-Cβ atom to the center of the green blobs indicated by green arrows in (**a**) versus dataset. The top and bottom dashed lines represent the tabulated distances Cβ-Sγ and Cβ-Oγ in cysteine and serine residues, respectively.

While the backbone structure remains unchanged, the difference Fourier maps, *F*_*obs*,*n*_ - *F*_*obs*,*1*_, reveal side-chain movements and specific radiation damage throughout the entire structure as accumulated absorbed dose increases. Signs of specific damage were not observed in the catalytic tetrad Tyr48, Lys77, His110 and Asp43^42^ (Fig. 2), which consistently agrees with the similar experimental *k*_cat_ values measured with the non-irradiated and irradiated enzyme. No conformational changes were observed at any irradiation dose in the other residues forming the active site of the protein except for the remarkable alteration on Cys298, which was developing upon irradiation (Fig. 3). This residue has been reported to regulate the kinetic and inhibition properties of the enzyme although it does not participate in the catalysis^13,14,16^.

Three different structural changes, seen as differential electron densities (blobs) in the difference Fourier maps at a resolution of 1.17 Å (Fig. 3a), can be observed in Cys298 as absorbed dose increases: a loss of electron density (red blob) accounting for the disorder and desulfuration induced by the irradiation, a small increase of the electron density (green blob pointed by blue arrows) compatible with an induced movement of the residue towards this region (Supp. Fig. 2), and the emergence of a positive electron density (green blob pointed by green arrows) at a distance of 1.47 Å from the Cβ-Cys298 atom for map D20-D1. This latter positive electron density is also observed in Fo-Fc electron density maps with progressively increasing absorbed dose (Fig. 3b). The maximum value of this positive electron density in the Fo-Fc map at the highest-dose dataset D20 is placed at a distance of 1.46 Å from the Cβ-Cys298. This distance falls well within the typical covalent C−O distances in both difference Fourier and Fo-Fc maps, suggesting that the new electron density can be explained by the capture of an oxygen atom from the solvent (probably from OH·) by a radicalized intermediate species. This process would effectively change the Cys298 residue present in the native form to a serine residue. The refinement of a serine and cysteine sharing the same Cβ at the 298 position, which is the model with the minimum number of refined variables to avoid over-refinement, leads to unrestrained distances between Cβ atom and Oγ and Sγ atoms of 1.41 ± 0.08 Å and 1.80 ± 0.03 Å, respectively (Fig. 3c). These distances are in excellent agreement with the tabulated values of 1.417 Å and 1.801 Å, respectively^43^. Moreover, the unrestrained distance between Cβ and Oγ atoms is constant for all datasets taken at different doses (Fig. 3e), suggesting that the bond is stable and thus the residue in this state can properly be identified as serine. Sγ-Cys298 and Oγ-Ser298 are well-defined atoms, as hinted by their atomic displacement parameter (ADP) values (12.24 Å^2^ and 15.64 Å^2^, respectively), which are similar to that of the Cβ atom (14.67 Å^2^).

The refined free occupancies for Sγ-Cys298 and Oγ-Ser298 at the maximum absorbed dose (dataset D20) are 0.68 and 0.25, respectively. As the overall occupancy for both species is lower than one, further minor concurrent species can be hypothesized to be coexisting with either a cysteine or a serine in the 298 position. Same hypothesis can be proposed from the evolution with absorbed dose of the electron density peaks in the difference Fourier maps (Fig. 3d). The sum of the maximum intensity of the blobs attributed to the cysteine desulfuration (Fig. 3d, pink circles) and the blob assigned to the Oγ capture (Fig. 3d, green squares) is consistently less than one for all datasets (Fig. 3d, gray triangles) and increasing with dose, suggesting that these putative extra species in residue 298 would be present in all the doses and that these are created by the X-ray irradiation (Fig. 3d, gray triangles).

### Liquid chromatography tandem mass spectrometry reveals C298S and C298A modifications

**Figure 4:**
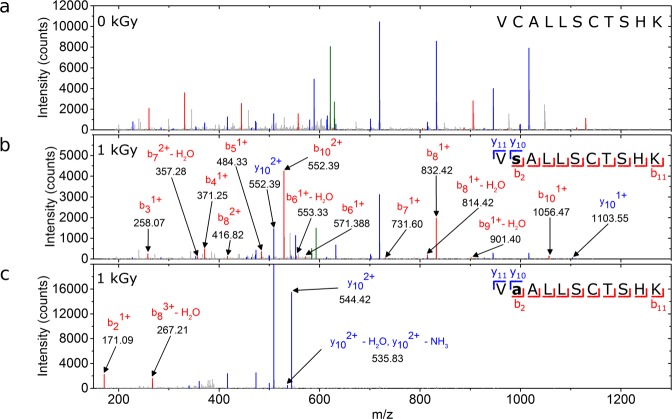
LC-MS/MS analysis. LC-MS/MS spectra for non-irradiated protein (**b**) and for the irradiated protein at 1 kGy subjected to modifications at position 298 from cysteine to serine (**b**) and alanine (**c**). Characteristic peaks from the peptide fragmentation are colored in red (bn), blue (yn) and green (precursor). Non-characteristic peaks from the sequence are marked in gray. For clarity, only peaks that contain information about modifications are labeled. Only peaks above 1% of the base peak and with match tolerance of 0.6 Da are represented.

Specific changes in Cys298 were also checked by liquid chromatography tandem mass spectrometry (LC-MS/MS) for the non-irradiated (0 kGy) and for the 1-kGy and 17-kGy irradiated proteins (Fig. 4 and Supp. Fig. 3). The Cys298-containing peptides were selected after trypsin digestion and fragmented into smaller ionized peptides. As expected, non-irradiated samples showed no modifications at any residue position (Fig. 4a). At an irradiation level matching the maximum activity peak of hAR (1 kGy), only two different modifications were detected along the peptide sequence: C298S and C298A (Fig. 4b). Over-irradiated samples at 17 kGy showed a C303A modification in addition to the C298S and C298A modifications (Supp. Fig. 3), which points to desulfuration of Cys303 by radiation damage. LC-MS/MS measurements thus are consistent with the C298S modification seen in MX experiments in irradiated samples, and suggest that the C298A modification is one of the extra species hypothesized at the 298 position in MX experiments. We do not discard that other species already reported in literature, such as sulfenic acid, could also be present^44,45^.

### Modeling of the active site with and without inhibitor shows why inhibitors are not effective in the activated form

**Figure 5:**
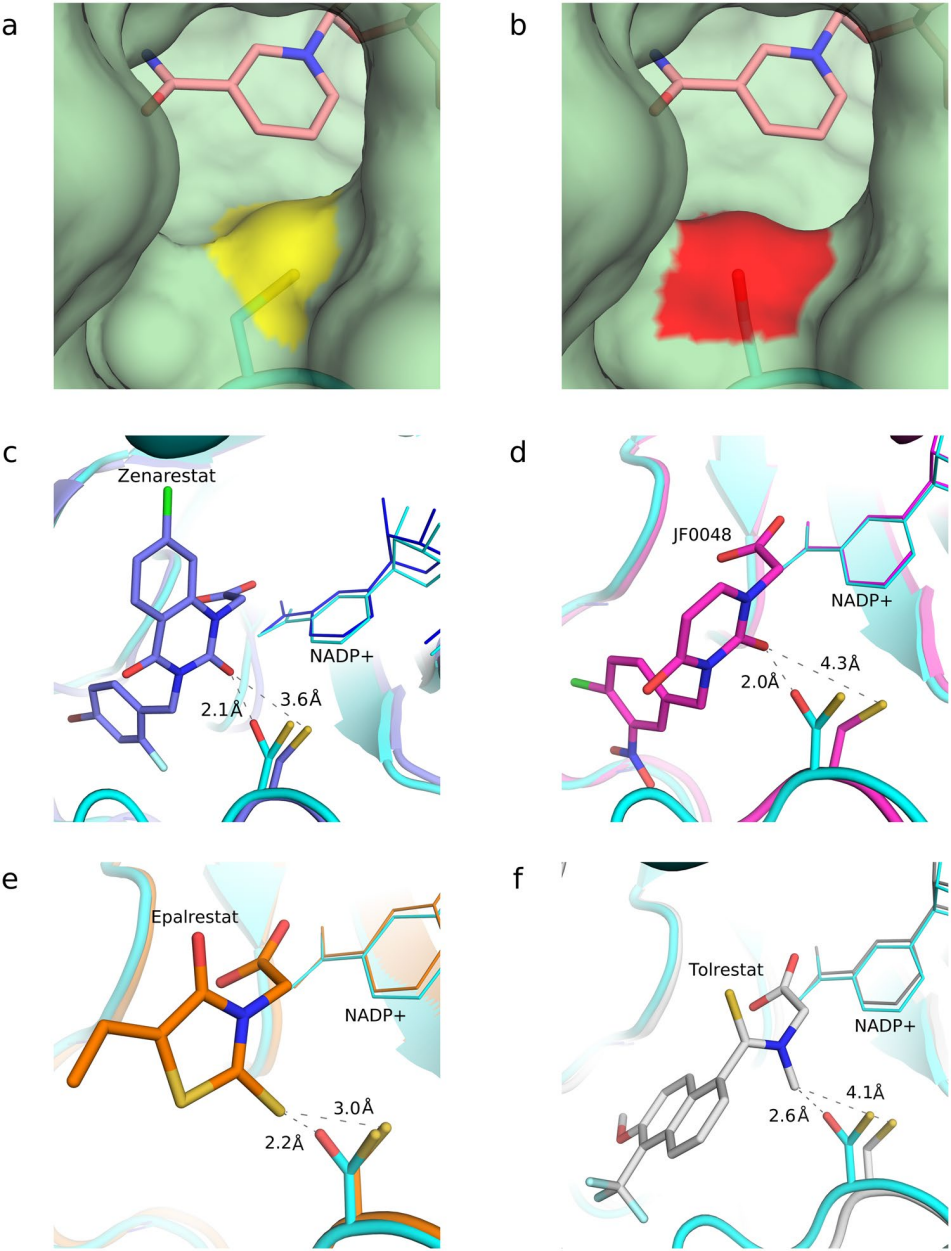
Changes on the topology of the hAR pocket entry channel and steric collisions of activated hAR with inhibitors. The conversion of Cys298 into Ser298 modifies the entrance of the substrate-binding pocket. (**a**) Entrance of the binding pocket of the native form of hAR. The sulfur atom of the Cys298 residue is marked in yellow. (**b**) Entrance of the binding pocket of the irradiated structure, which shows the Oγ-Ser298 atom, marked in red. In both cases, the nicotinamide ring of the cofactor is shown in pink color. (**c**–**f**) Interaction of the studied inhibitors with Cys298 and Ser298. The structure of the D20 dataset (in cyan) is superimposed with the crystallographic structures of hAR including the inhibitors Zenarestat (**c**, purple, PDB 1IEI), JF0048 (**d**, magenta, PDB 4XZH), Epalrestat (**e**, orange, PDB 4JIR) and Tolrestat (**f**, gray, PDB 1AH3). In all cases the inhibitors, designed against the native form, show severe steric collisions with Oγ-Ser298 but not with Sγ-Cys298. Sulfur atoms are displayed in yellow whereas oxygen atoms are displayed in red.

Although Cys298 is not a catalytic residue in hAR, the conversion of this residue into a Ser or Ala is key in the functional properties of hAR and in the sensitivity to ARIs. This is explained by the localization of Cys298 between the substrate-binding pocket and the active site, where NADP+ and the catalytic tetrad are found. Cys298 is therefore controlling the substrate accessibility to the active site. The crystallographic structure reveals that the conversion of the Cys298 residue (Fig. 5a) into a serine (Fig. 5b) changes the position of the atom in the γ position of the residue and the type of element, thus modifying the entrance to the substrate-binding pocket. The protein-ligand interaction may be consequently affected. This view is consistent with the enzymatic results described above, which show an increase in the K_M_ value and a reduction in the potency of the tested inhibitors.

The steric changes in the entrance of the binding pocket severely hinder the interactions between hAR and the inhibitors originally designed against the native form, including a cysteine in the 298 position (Fig. 5). The alignment between the D20 dataset crystallographic structure and that with the various inhibitors shows that the Sγ-Cys298 atom has a very low displacement (Supp. Table 3). The distances of the Sγ-Cys298 atom to the closest atom of the studied inhibitors can thus be compared, and are shown to be always above 3 Å. The native form is therefore sterically compatible with the inhibitors, as expected since they were designed against it as a target. On the other hand, in the irradiated protein where cysteine has been replaced with serine, the distance between the Oγ-Ser298 atom and the closest atom of the aligned inhibitors is much shorter and could lead to steric collisions that may affect their binding to the active site. Remarkably, Tolrestat, which had the highest inhibitory potency among the tested compounds on the irradiated enzyme (Fig. 1), also shows the longest distance between its closest atom and the Oγ-Ser298 atom, 2.6 Å. This fact reinforces the view that the activated form of hAR interacts less efficiently with inhibitors due to the conversion of Cys298 into Ser298 under oxidative stress conditions.

### The activation of hAR is mediated by the free electrons present under oxidative stress conditions

**Figure 6:**
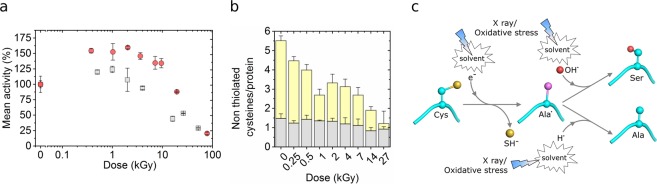
Indication that electrons are the main agent for the activation of hAR and proposed mechanism. (**a**) hAR activity in the presence of 10 mM uridine (red circles) and in the presence of 10 mM uridine plus 40 mM sodium nitrate (gray circles) as a function of irradiation dose. (**b**) Number of non-thiolated cysteines per protein molecule as a function of dose, as measured by the Ellman’s assay. Accessible cysteines (in the absence of urea) are shown in gray, and buried cysteines (revealed in the presence of 8 M urea) are shown in yellow. (**c**) Scheme of the proposed mechanism for the C298S and C298A conversion induced by X-ray irradiation or oxidative stress. Carbon atoms are colored in cyan, carbon radicals in purple, oxygen atoms in red, and sulfur atoms in yellow. Hydrogen atoms are not shown for clarity.

Specific radiation damage in MX experiments at cryogenic temperatures results essentially from the action of secondary electrons that are generated by the photoelectrons ejected from water and organic molecules. The secondary electrons are highly mobile and can either be spread through the solvent or be tunneled into the protein structure through the main chain until they find electrophilic sites such as metal centers and acidic or sulfur-containing residues, especially cysteine^46^. The other reactive species generated from water radiolysis show low mobility at cryogenic temperatures and their effect on the structural damage can be neglected. To test whether the loss of the thiol group in Cys298 of hAR, observed by MX experiments at cryogenic temperature. can be attributed to electron capture as well in protein solution at room temperature, the relative activity of hAR was measured as a function of dose in the presence of different compounds with variable scavenging properties depending on the reactive species (Fig. 6).

Uridine has recently been reported effective only against radiation damage at room temperature and ineffective at cryogenic temperatures^36^. As the electrons are the main mobile reactive species at cryogenic temperatures^47^, this suggests that uridine is only effective against ROS and not against free electrons and therefore it may be a probe to test the action of free electrons in the activation process. The relative activity of hAR in the presence of 10 mM uridine (Fig. 6a, red dots) is similar to that of hAR without a scavenger (Fig. 1a). In both cases the relative activity increases by ~50% at 1 kGy and follows a sigmoidal decay at higher doses. Uridine is thus not preventing the production of the activated form of hAR under X-ray irradiation by the action of its ROS scavenging properties. Nevertheless, uridine still prevents global radiation damage to hAR since the dose at which the enzymatic activity is reduced to the half with respect to the maximum relative activity is increased from 10.9 ± 0.8 kGy without uridine (Fig. 1a) to 26 ± 2 kGy with 10 mM uridine (Fig. 6a). The uridine protective effect is also observed in the conservation of the relative radius of gyration of hAR in solution as measured by SAXS (Supp. Fig. 4). To further assess the action of electrons in the activation of hAR, sodium nitrate, a well-known electron scavenger^48^, was added to the solution of hAR with 10 mM uridine. By adding 40 mM sodium nitrate, the relative activity of hAR increased only by 25% at 1 kGy with respect to non-irradiated sample, and decreased at higher doses with a sigmoidal behavior (Fig. 6a). Remarkably, this activity increase in the presence of sodium nitrate (~25%) was significantly lower than that obtained with uridine only, which is fully compatible with the electron capture as the main source of activation of hAR. Electron capture is also consistent with the quantity of non-thiolated cysteines per hAR molecule as a function of dose measured by the Ellman’s assay (Fig. 6b). The number of buried non-thiolated cysteines per molecule decays as a function of dose, whereas the number of cysteines accessible to the Ellman’s reagent per molecule remains approximately constant.

The activities at different doses and scavengers allow drawing an hypothesis of the mechanism for the conversion of residue Cys298 into Ser298 and to Ala298 based on the cysteine desulfuration involving highly mobile electrons either from the solvent or tunneled through the backbone and absorbed by hAR electrophilic centers such as sulfur atoms^46,49,50^. This hypothesis is reinforced by the desulfuration of non-exposed cysteines observed in our MX experiments under cryogenic conditions, in which electrons are essentially the only mobile reactive species^51^. Whichever the origin of the free electrons is, after the absorption by the cysteine and the loss of the thiol group, residue 298 enters in an excited, metaestable state^49,50^ which can bond either to a hydroxyl group to form a serine or to a hydrogen radical to form an alanine (Fig. 6c). At this stage we cannot exclude other products generated by alternative or complementary mechanisms.

## Discussion

The activated form of hAR, as found under oxidative stress conditions *in cellulo*, has been reported to have different kinetic constants than the native form, in particular a much larger K_M_ value and a more stable *k*_*cat*_ value^13,14,52^, and to reduce the efficacy in clinical trials of ARIs developed and tested *in vitro*^13,15,52^. Here we have reproduced this characteristic behavior of the activated hAR in irradiated native protein solution. X rays are massively absorbed by the ~1000 mg/mL water content of the solution, rather than the protein at a concentration of 2.3 mg/mL, and they induce the same reactive species as those present under oxidative stress conditions^21,29^, where the activated form has been found^10,12^.

A generic unwanted side effect of using X-ray radiation to induce reactive species is to produce global structural changes in the protein as a consequence of the primary interaction of X rays with the protein atoms. These structural changes, which would not be produced in cells under oxidative stress conditions, could in principle limit the capacity of irradiation methods to mimic the catalytic behavior under stress conditions. In this respect, our data do not discard conclusively that there are no such global structural changes at doses up to 1 kGy, when the values of the kinetic constants and the reduced potency of ARIs suggest a significant activation of hAR. However, our data support that, in case there was such global radiation damage on the protein, it is not affecting the catalytic reaction, as the *k*_*cat*_ value does not change at this dose level. Contrarily, the existence of relevant specific changes is supported by the dramatic increase in the K_M_ value, indicating that the interaction with the substrate is severely affected. The hypothesis that better matches this behavior of the kinetic parameters is a change in the d,l-glyceraldehyde substrate binding pocket. The Cys298 residue, which lies at the entrance of the binding pocket, is an excellent candidate for this change. This hypothesis is consistent with previous literature, which shows that the mutation of Cys298 increases the K_M_ value by one order of magnitude without severely affecting the k_cat_ value^13,14,52^.

As radiation damage is expected to induce a loss of the catalytic activity, it is noticeable that the activity of hAR in solution increases by 50% when irradiated up to 1 kGy (Fig. 1a). This moderate increase in the activity can be explained by the saturation at higher concentrations of the d,l-glyceraldehyde substrate which in turn leads to the loss of substrate inhibition, as indicated by the dramatic increase of the K_M_ value (Fig. 1c). Consistently, the activity blockade by the ARIs, which also bind to the d,l-glyceraldehyde pocket, is also reduced (Fig. 1b).

The structural studies at atomic resolution are in line with our proposed hypothesis that the presence of a serine or alanine in residue 298 instead of cysteine in an oxidative stress environment is causing the activation of the hAR. Other species reported to oxidize Cys298 such as sulfenic acid can not be excluded^45^ to intervene in the activation. Irradiation of the native form induces the same structural changes at atomic level as those suggested for the activated form^12,14,37,53^, that is, the modification of the residue 298 while the catalytic tetrad, as well as all the other residues around the binding pocket, remain unaltered (Fig. 2). According to the results presented here, the activated form differs structurally from the native form by the replacement of the Cys298 residue by Ser298 and, to a lesser extent, by Ala298. We can not exclude further changes at this position which might as well modify the interaction with ARIs. The modification of this key residue affects the entrance to the substrate-binding pocket, resulting in a altered binding of the substrate and of the inhibitors, which finally leads to a reduction in the inhibitory potency of the drugs initially designed to bind to the native form of hAR.

Incidentally, we note that more substrates other than d,l-glyceraldehyde might be tested. However, given that glyceraldehyde is one of the smallest substrates for hAR, the accessibility of this substrate to the binding pocket is high, as it can be accommodated into the altered binding pocket more easily than other common, larger substrates such as glucose, xylose or 4-hydroxynonenal, among others. Therefore, it can be expected that the potency loss of the inhibitor compounds observed using glyceraldehyde as a substrate will also appear at least in a similar extent using other common substrates under the same oxidative stress conditions.

The conversion of the residue Cys298 into Ser298 and Ala298, and possibly other species, is hypothesized to be triggered by the presence of free electrons available under X-ray irradiation, as indicated by the lesser increase of activity upon irradiation in the presence of sodium nitrate (a known free electron scavenger) compared to the higher increase when adding uridine (a ROS scavenger) in the solution. The free electrons might be either induced in the solvent or tunneled into the protein structure through the protein main chain.

In spite of the degradation of the modified proteins and the continuous expression of the native form in living cells, the described activated forms prevail in cells from diabetic patients as the proteins newly expressed are modified recurrently under permanent oxidative stress conditions. Therefore, we propose that ARIs should be designed against the activated forms of hAR, here generated by X-ray irradiation, which show a similar behavior against ARIs to the activated forms of hAR found under hyperglycemic conditions^10,11,20^. The attempts to develop ARIs employing the native, genome coded form of hAR risk to fail when tested in real diabetic tissues or patients, as it happened in many previous cases^10,11^. In particular, ARIs should be designed excluding the interaction of the inhibitor with Sγ in Cys298 and taking into account the Oγ in residue Ser298 appearing in the activated form. In order to best mimic the physiological situation, we therefore suggest using irradiated protein in high-throughput drug discovery and drug development studies, including *in vitro* testing, to complement studies in site-directed mutants and to increase the rate of success of ARIs in subsequent clinical trials. The crystal structure of the irradiated hAR presented in this study (PDB: 6F8O), which includes the double modification C298S and C298A with respect to the native form, can also be employed as a target for an effective rational in silico drug discovery. The structure would allow taking into account and modeling the interactions between a lead molecule and the distinct active-site pocket of active hAR, especially near the residue 298. Furthermore, since the mechanisms that involve reactive species are common in oxidative stress and/or irradiation conditions, the controlled method of irradiation with X-rays presented in this work could become an instrumental approach to study other proteins that are also exposed to oxidative stress environments, like those related to cancer, Parkinson, Alzheimer or inflammatory diseases.

## Methods

### Protein purification and crystallization

The hAR cDNA obtained from Alexandra Cousido-Siah and Alberto Podjarny (IGBMC-CNRS-Univ. Strasbourg) was cloned into the pET-15b expression vector. The expression of the His6-tagged protein in *Escherichia coli* strain BL21 (DE3) pLysS was induced at 20 °C by 1 mM IPTG at OD600 0.8–1 and cells were incubated for 16 h in a 2 L culture. Then cells were disrupted by sonication and centrifuged at 4 °C in 20 mM Tris-HCl pH 8.0, 250 mM NaCl, 5 mM imidazole buffer. The pellet was discarded and the supernatant was loaded in a nickel ion affinity chromatography column. The hAR was eluted in an imidazole gradient (5–250 mM). Then, thrombin cleavage of the His-tag was carried out overnight at 277 K. The buffer of the cleaved protein was changed to 50 mM ammonium citrate pH 5.0. The hAR crystals were grown by the hanging drop vapor diffusion method. Ten μL of protein at 30 mg/mL with a NADPH:protein ratio of 2:1 was mixed with 10 μL of 50 mM ammonium citrate pH 5.0, 10% (w/v) polyethylene glycol (PEG) 6000 and 1 mM DTT. The solution was equilibrated at 4 °C for a week against a 500 μL reservoir, consisting of 20% PEG 6000 and 120 mM ammonium citrate, pH 5.0. After drop equilibration, nucleation was induced by seeding at room temperature. Crystals grew until its maximum size (around 0.8 mm long) in three days and finally they were transferred into a cryoprotecting solution consisting of 85 mM ammonium citrate pH 5.0 and 40% PEG 6000 prior to be loop-mounted and stored in liquid nitrogen.

### Protein irradiation in solution for the enzymatic assays

For a given sample, 3 μL at 2.3 mg/mL in 20 mM Tris-HCl, 250 mM NaCl pH 8.0 were introduced inside a polyimide cylindrical capillary of 1 mm internal diameter and 0.025 mm wall thickness. The ends were sealed with vacuum grease to prevent liquid evaporation. The capillary was placed in a magnetic base compatible with the BL13-XALOC goniometer (Supp. Fig. 5). Homogeneous irradiation was ensured by scanning the whole sample volume at uniform speed using a top-hat rectangular-shape X-ray beam of 200 × 180 μm^2^ (Supp. Fig. 5). The photon flux at sample was measured prior to each experiment by a calibrated Si PIN diode. The photon energy was set to 12.661 keV (0.979 Å).

### Enzymatic assays

The hAR activity was determined with a Varian Cary 400 (UV/VIS) spectrophotometer at 25 °C by monitoring at 340 nm the decrease of NADPH in 0.6 mL of 0.1 M sodium phosphate buffer pH 7.4, containing 2 μL of irradiated or native enzyme, 0.2 mM NADPH and 10 µM–30 mM d,l-glyceraldehyde. For inhibition assays, activity was measured under the same conditions, in the presence of 6 mM d,l-glyceraldehyde. Concentrated stocks of inhibitors were prepared in DMSO and diluted in the reaction mixture to reach a final compound concentration close to the IC_50_ value in a final DMSO concentration of 1% (v/v). Control activity was performed in the presence of 1% (v/v) DMSO. All the experiments were performed at least in triplicate, using for each activity an independently irradiated protein sample.

From the slope of the absorbance vs time graph, the variation of absorbance per second was obtained, which was then converted to specific activity using NADPH molar extinction coefficient (ε = 6,220 M^−1^ cm^−1^), enzyme concentration and sample volume applied and protein molecular weight (35.9 kDa). Specific activities obtained for native protein at substrate concentrations ranging from 10 μM to 30 mM were fitted to the substrate inhibition equation:

$$V=\frac{{V}_{max}\ast [S]}{\,{K}_{M}+[S]\ast (1+\frac{[S]}{Ki})\,}$$

In the case of 1kGy irradiated protein, as no significant substrate inhibition was observed in the tested concentrations, data was fitted to the Michaelis-Menten equation:

$$V=\frac{\,{V}_{max}\ast [S]\,}{{K}_{M}+[S]}$$

### MX data collection

Diffraction data collections at 100 K were performed in the BL13-XALOC beamline at the ALBA synchrotron using a Pilatus 6 M photon-counting detector (DECTRIS, Baden, Switzerland). The beam was set at 13.750 keV photon energy (λ = 0.9017 Å) and the sample-detector distance was adjusted to collect the datasets at a resolution of 0.90 Å. The attenuated photon flux during data collections was 1.2 × 10^11^ photons/s as measured using a calibrated Si PIN diode at the sample position. The beam profile had a top-hat circular shape and was defocused to 190 µm in diameter as measured by a Ce:YAG fluorescent screen at the sample position (Supp. Fig. 6). Twenty complete data sets (D1-D20) of 800 images each were collected with an angle increment of 0.25 deg and 0.1 s exposure time per image. The crystals were irradiated between datasets using a beam with a photon flux of 6.3 × 10^11^ photons/s to speed-up radiation damage effects. The total oscillation angle was identical for all burns and data collections.

### MX data processing and structure refinement

All twenty datasets collected in the MX experiments were indexed and integrated using XDS^54^ in P21 space and cell parameters similar to those previously reported. The datasets were scaled and the structure factors generated using AIMLESS^55^ and TRUNCATE^56^, respectively. The resolution cutoff for each dataset was chosen so that R_merge_ was around 30% in the last resolution shell. Following this criterion, the I/σI in the last resolution shell ranged between 3.6 and 4.7 for all the datasets (Supp. Table 1). The initial phases to solve the structure of the D1 dataset were calculated from PDB entry 2J8T^57^ after removal of water molecules and alternative conformations with Phaser-MR^58^. To reduce the model bias, all the atom coordinates from the initial model were randomly shifted by 0.15 Å using PDBSET and the Cys298-Sγ and Cys298-Cβ atoms were removed. The initial phases for D2-D20 structures were extracted in the course of a refinement process based on the previously solved structure after the removal of water molecules with a B-factor > 35 Å^2^. All the structure refinements were carried out using SHELXL^59^. The examination of the density maps and the manual rebuilding of the model were done using Coot^60^. The refined parameters included atomic coordinates, atomic displacement parameters (ADP), and atomic occupancy. The bond length standard uncertainties were computed by inversion of the full normal matrix with SHELXL. No riding hydrogen atoms were included in the model. The difference Fourier maps were built using reflections between 50 Å and 1.2 Å resolution and were Wilson scaled together via SCALEIT^61^. The difference Fourier maps (F_obs,n_ - F_obs,1_, α_calc,1_) were calculated between each dataset n and the initial dataset using the phases of the fresh, not previously irradiated dataset, α_calc,1_. The complete data collection and refinement statistics are listed in Supp. Tables 1 and 2 respectively. The crystal structures were deposited at the Protein Data Bank with PDB IDs 6F7R, 6F81, 6F82, 6F84 and 6F8O for datasets D1, D5, D10, D15 and D20, respectively.

### MX structure alignment

The alignment between the crystallographic structures were carried out by LSQKAB using the method described by Kabsch^62^ between residues Ser2 and Phe311. The root mean square deviation (RMSD) of the C298 residue and that of the whole structure excluding the disordered terminal residues (Ser2 to Phe311) were calculated for the Cα, main chain, side chain and all atoms (Supp. Table 3).

### SAXS data collection

SAXS data from B21 Beamline (Diamond Light Source, Didcot, United Kingdom) was obtained using a Pilatus 2 M photon-counting detector (DECTRIS, Baden, Switzerland) and a camera length of 4.014 m. The beam at the sample position was 4 mm (horizontal) × 0.96 mm (vertical) and had an incident flux of 8.5 × 10^10^ photons/s at 12.4 keV (λ = 0.999 Å). The hAR samples were measured in 100 mM sodium phosphate buffer, pH 7.4, at a concentration of 2.7 mg/ml. For the samples containing uridine, a 10x stock solution of the additive was prepared in the same buffer and ten-fold diluted to a final concentration of 10 mM prior to the measurements. All samples were analyzed in a cylindrical quartz capillary of 1.6 mm internal diameter with a wall thickness of 0.1 mm, held at 283 K under vacuum. Multiple frames of 10 s exposure time were recorded. The data were collected by using the BioSAXS sample changer robot^63^. The experiments were performed in static mode and without beam attenuation to speed-up radiation damage on the sample. The SAXS experiments with samples containing uridine were performed under the same conditions.

### SAXS data analysis

Radius of gyration (R_g_) was determined with the Guinier approximation^64^ by using the PRIMUS software^65^ included in the ATSAS package^66^. Radiation damage was monitored by the normalized radius of gyration calculation, R_g_/R_g0_, where R_g_ is the radius of gyration at a given dose and R_g0_ is that obtained in the first data frame. An increase of more than 10% in the normalized radius of gyration is considered indicative of significant global structural radiation damage.

### Dose calculations

The dose absorbed by the samples in the enzymatic and SAXS experiments was calculated by using the Lambert’s Law:

$$D=\frac{{F}_{in}\, < \,Trans\,{ > }_{container,1}\,E\,t < \,Abs\,{ > }_{sample}}{m}$$

where *F*_*in*_ is the incoming photon flux, *E* is the photon energy, t is the accumulated exposure time, m is the mass of the irradiated sample, <*Trans*>_*container*,*1*_ is the average transmission coefficient for one side of the container and <*Abs*>_*sample*_ is the average absorption coefficient for the irradiated sample.

For these experiments <*Trans*>_*container*,*1*_ was calculated following:

$$ < \,Trans\,{ > }_{container,1}=\frac{1}{2{R}_{ext}}{\int }_{-{R}_{ext}}^{{R}_{ext}}\exp (\,-\,\mu \rho d/2)dx$$

where µ is the attenuation coefficient of the container at the used photon energy, *ρ* is the density of the container, R_ext_ is the external radius and *d* is the beam path length through the container. <*Trans*>_*container*,*1*_ was calculated to be 0.992 for the polyimide container (µ = 1.494 cm^2^/g and ρ = 1.425 g/cm^3^) and 0.690 for the quartz container (µ = 9.804 cm^2^/g and ρ = 2.648 g/cm^3^) (Supp. Fig. 7).

In the case of enzymatic experiments, <*Abs*>_*sample*_ was calculated from:

$$ < \,Abs\,{ > }_{sample}=1-\frac{1}{ < \,Trans\,{ > }_{container,1}^{2}}\frac{1}{2{R}_{in}}{\int }_{-{R}_{in}}^{{R}_{in}}\frac{{F}_{out}(x)}{{F}_{in}}dx$$

where F_out_ is the output photon flux, <*Trans*>_*container*,*1*_ is taken from the polyimide container and *R*_*in*_ is the internal radius of the container. *F*_*in*_ and *F*_*out*_(x) were measured experimentally by scanning the capillary the x direction with a calibrated Si PIN diode (Supp. Fig. 8). For enzymatic experiments <*Abs*>_*sample*_ was calculated to be 0.256.

Since the protein concentration for the SAXS experiments was very low (~75 µM), the protein solution was approximated to be only water (µ = 2.673 cm^2^/g and ρ = 1 g/cm^3^). With this assumption, <*Abs*>_*sample*_ can be calculated through:

$$ < \,Abs\,{ > }_{sample}\approx 1-\frac{1}{2{R}_{int}}{\int }_{-{R}_{int}}^{{R}_{int}}\exp (\,-\,\mu \rho D)dx$$

where *D* is the beam path length through the sample. For SAXS experiments <*Abs*>_*sample*_ was calculated to be 0.282. All the attenuation lengths µ and density *ρ* values were extracted from the XCOM Photon Cross Sections Database (https://physics.nist.gov/cgi-bin/Xcom/xcom2). The average dose in the exposed region (AD-ER) for the MX experiments was calculated with RADDOSE-3D^67^.

### Inhibitor compounds

Zenarestat (3-(4-bromo-2-fluorobenzoyl)-7-chloro-3,4-dihydro-2,4-dioxo-1(2H)-quinazolineacetic acid) was generously provided by A. Podjarny. JF0048 (2-(3-(4-chloro-3-nitrobenzyl)-2,4-dioxo-3,4-dihydropyrimidin-1(2H)-yl)acetic acid) was a gift from A.R. de Lera (Univ. Vigo), and was synthesized as described previously^31^. Tolrestat was a kind gift of Prof. T.G. Flynn (Queen’s University, Kingston, Canada). Epalrestat was purchased from Sigma-Aldrich (reference SML0527).

### Liquid chromatography– tandem mass spectrometry (LC-MS/MS)

Proteins were manually digested with trypsin (Sequencing grade modified, Promega). Briefly, proteins were reduced by treatment with a solution of 20 mM DTT in 50 mM NH_4_HCO_3_ for 30 min at 56 °C, and then alkylated by treatment with a 50 mM solution of iodine acetamide for 30 min at room temperature. Proteins were digested overnight, at 37 °C with 100 ng of trypsin. Tryptic peptides were cleaned up with C18 homemade stage tips. Eluted peptides were dried in a vacuum centrifuge. The dried-down peptide mixtures were re-suspended in 1% formic acid and analyzed in a nanoAcquity liquid chromatographer (Waters) coupled to a LTQ-Orbitrap Velos (Thermo Scientific) mass spectrometer. Peptides were trapped on a Symmetry C18TM trap column (5 μm particle size, 180 μm × 20 mm; Waters), and were separated using a C18 reverse phase capillary column (ACQUITY UPLC M-Class Peptide BEH column; 130 Å, 1.7 μm, 75 μm × 250 μm, Waters). The gradient used for the elution of the peptides was 1 to 40% in 30 min, followed by a gradient from 40 to 60% in 5 min (A: 0.1% formic acid; B: 100% acetonitrile, 0.1% formic acid), with a 250 nL/min flow rate. Eluted peptides were subjected to electrospray ionization in an emitter needle (PicoTipTM, New Objective) with an applied voltage of 2000 V. For each irradiated, peptide masses (m/z 300–1700) were analyzed in data dependent mode where a full Scan MS was acquired in the Orbitrap instrument with a resolution of 60,000 FWHM at m/z 400. Up to the 15 most abundant peptides (having a minimum intensity of 500 counts) were selected from each MS scan and then fragmented in the linear ion trap using CID (38% normalized collision energy) with helium as the collision gas. The scan time settings were: Full MS: 250 ms (1 microscan) and MSn: 120 ms. The scan time settings were 250 ms (2 microscan) for full MS, and 300 ms for MSn. The raw data files were collected and generated using Thermo Xcalibur (v.2.2). All the steps in the LC-MS/MS measurements were performed at the Plataforma de Proteòmica of the Parc Científic de Barcelona.

### Analysis of non-thiolated cysteines

The reduction state of cysteines was assayed following the Pierce procedure for quantifying sulfhydryl groups based on molar absorptivity. Briefly, the reduced cysteines are detected by their reaction with 5,5′-dithiobis-(2-nitrobenzoic acid) (DTNB)^68^, which gives a product with a maximum absorbance at 412 nm. The reaction buffer used in the analysis of exposed cysteines consisted of 0.1 M sodium phosphate, 1 mM EDTA, pH 8.0. The reaction buffer used in the analysis of total cysteines added 8 M urea. The DTNB solution was prepared by mixing 4 mg of the reagent with 1 mL of reaction buffer (with or without urea). Two μL of the irradiated or native protein were mixed with 2.5 μL of reagent preparation and 7.5 μL of reaction buffer, giving a final reaction volume of 10 μL. The reaction was left at room temperature until the measure was stable (from 15 min to 5 h). Experiments were carried out in triplicate for each dose and for the buffer with or without urea, and used native protein as a control for each dose measure. Absorbance at 412 nm was monitored with a Nanodrop 2000 (Thermo Scientific).

The number of reduced cysteines per protein molecule was calculated for each dose and the buffer with or without urea using the Lambert’s law corrected to take into account the protein concentration:

$$Cy{s}_{red}/protein=\frac{Ab{s}_{412nm}}{\varepsilon \ast l\ast [Protein]}$$

where ε is the extinction coefficient of DTNB and *l* is the path length, which was 0.1 cm in our setup. The values of the molar extinction coefficients were assumed to be ε = 14,150 and ε = 14,290 M^−1^·cm^−1^ for the native and the denaturing conditions, respectively.

## Supplementary information

Supplementary Information

## Data Availability

The data generated by the enzymatic assays, the liquid chromatography-tandem mass spectrometry measurements and the crystallography and small angle scattering experiments, including the raw images which support the findings of this study, are available from the Zenodo repository with the identifier 10.5281/zenodo.1265630. The hAR structures obtained using datasets D1 (non-irradiated), D5, D15 and D20 (maximum irradiation) are uploaded on the PDB database (www.rcsb.org) with ID 6F7R, 6F81, 6F82, 6F84, 6F8O, respectively.
