# Supplementary material for: Efficacy of aldose reductase inhibitors is affected by oxidative stress induced under X-ray irradiation
Source: Sci Rep. 2019 Feb 28;9:3177. doi: 10.1038/s41598-019-39722-0 (PMC6395642; doi:10.1038/s41598-019-39722-0)
Supplement: Supplementary file 1 — Supplementary Information [file 41598_2019_39722_MOESM1_ESM.docx]

# Supplementary information

**Efficacy of aldose reductase inhibitors is affected by oxidative stress induced under X-ray irradiation**

Albert Castellví^a^^[[1]](#footnote-1)^, Isidro Crespo^a1^, Eva Crosas^a^^[[2]](#footnote-2)^, Ana Cámara-Artigas^b^, José A. Gavira^c^, Miguel A. G. Aranda^a^, Xavier Parés^d^, Jaume Farrés^d^, Judith Juanhuix^a^*.

^a^ Alba Synchrotron, carrer de la Llum 2-26, 08290 Cerdanyola del Vallès, Barcelona, Catalonia, Spain

^b^ Dep. of Chemistry and Physics, Universidad de Almería, 04120 Almería, Spain

^c^ Laboratorio de Estudios Cristalográficos, Instituto Andaluz de Ciencias de la Tierra, CSIC-Universidad de Granada, Avenida de las Palmeras 4, 18100 Armilla, Granada, Spain

^d^ Dep. of Biochemistry and Molecular Biology, Faculty of Biosciences, Universitat Autònoma de Barcelona, 08193 Bellaterra, Barcelona, Catalonia, Spain

* Corresponding author: juanhuix@cells.es, ORCID: 0000-0003-3728-8215





Supplementary Figure 1. Specific activity of hAR in presence of the inhibitors at different absorbed doses. Control activities in presence of vehicle (1% DMSO) are also displayed. As described in Materials and Methods, the inhibitors were solubilized in DMSO and diluted in the reaction mixture to 1% (v/v). Note that the presence of DMSO alters the activities of the vehicle control, acting as inhibitor of the hAR in native form, that is, at zero dose [1]. The inhibitory effect of DMSO is not known for irradiated hAR. To remove the effect of DMSO in activity assays, which adds complexity to the analysis, we normalize the data in Figure 1b with respect to the vehicle control at every dose. The raw activities shown in this figure can not be compared to the normalized activities of Figures 1a and 1c in the main text, which were obtained from samples without DMSO.

_
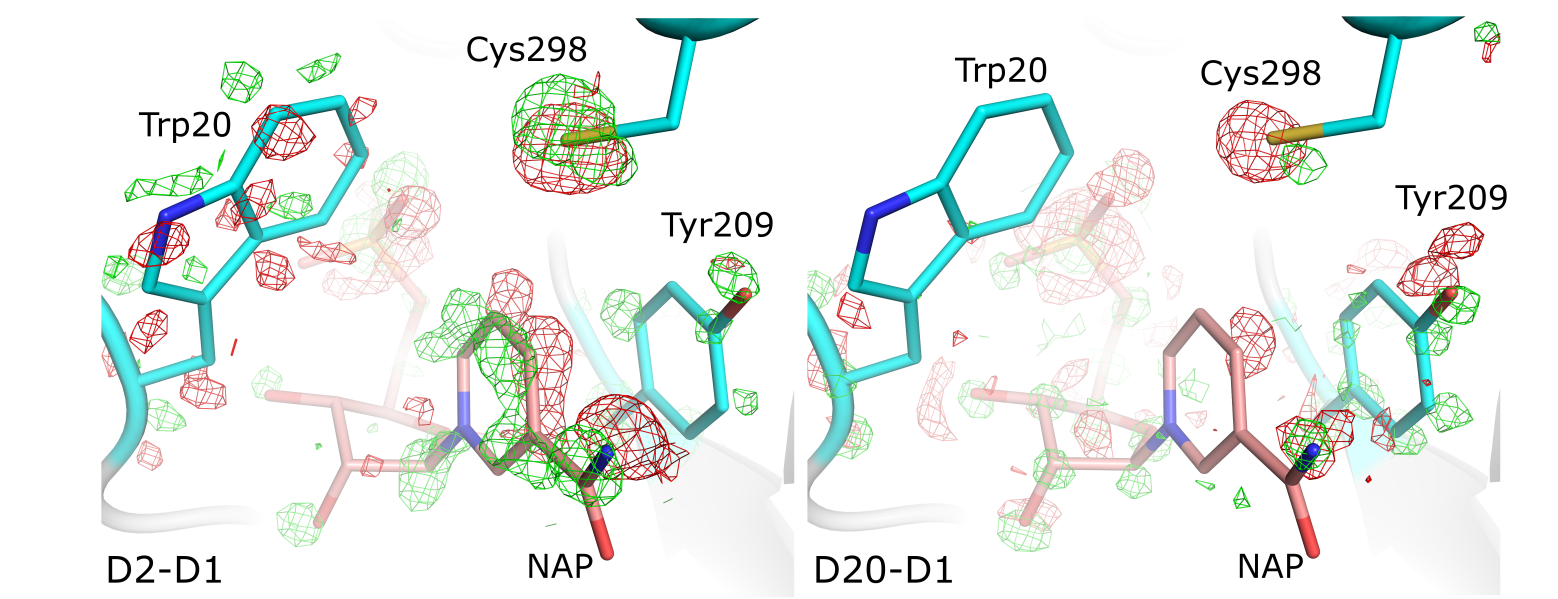
_

Supplementary Figure 2. Radiation induces movements in the vicinity of Cys298. Difference Fourier maps D2-D1 (a) and D20-D1 (b) in the vicinity of Cys298, calculated as in Figure 2 of the article. The D2-D1 difference Fourier map reveals structural changes over NADP^+^ (NAP), Trp20 and Cys298 (also slightly damaged), whereas the D20-D1 difference Fourier map reveals structural changes only over Tyr209. Both maps are contoured at σ = ±3.

**
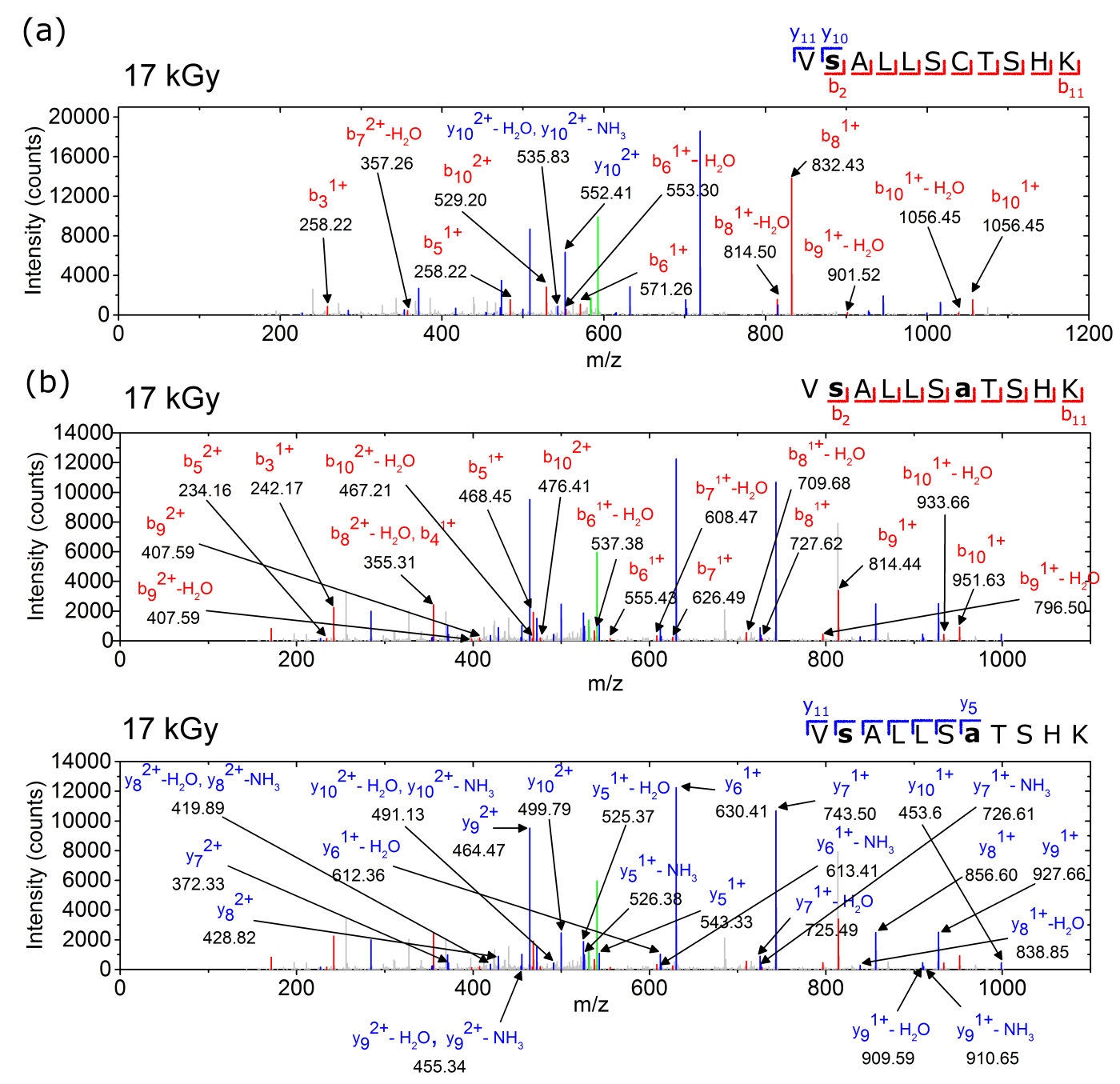
**

Supplementary Figure 3. LC-MS/MS analysis at 17 kGy. Two modifications were observed at an absorbed dose of 17 kGy in different Cys298-containing peptides: (a) peptide with the single C298S modification and (b) peptide with C298 and C303A modifications. Characteristic peaks from the peptide fragmentation are colored in red (b_n_), blue (y_n_) and green (precursor), and labelled in separate plots in (b) for clarity. Non-characteristic peaks from the sequence are marked in grey. For clarity, only peaks that contain information about the peptide modifications are labeled. Only peaks above 1% of the base peak and a matching tolerance of 0.6 Da are represented.


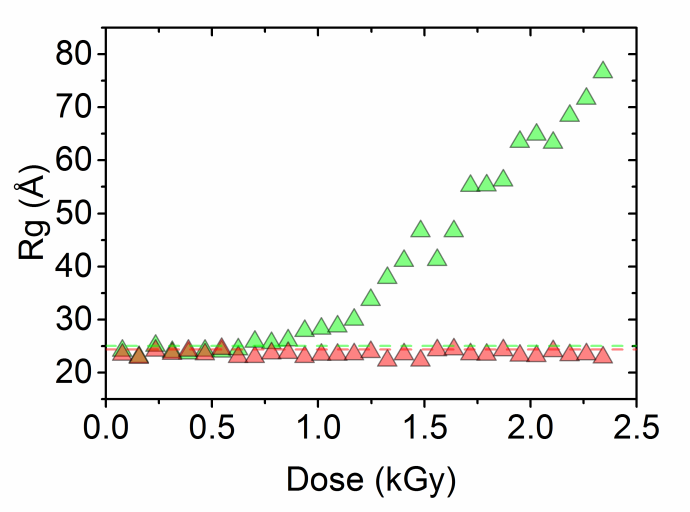


Supplementary Figure 4. Uridine prevents global radiation damage of hAR in solution at radiation doses over 1 kGy. Evolution of the normalized radius of gyration R_g_/R_g0_ of hAR in solution at a concentration of 2.3 mg/mL as a function of dose, as calculated in SAXS experiments performed in the beamline B21 at the Diamond Light Source. The hAR without any additive (green triangles) shows global radiation damage effects at absorbed doses above 1 kGy. The addition of 10 mM uridine to the hAR solution (red triangles) dramatically delays the global radiation damage to doses above 2.5 kGy. The dashed lines mark the initial radius of gyration incremented by 1 Å, above which the protein is considered damaged, unfolded or aggregated, according to Hopkins and Thorne, 2016 or Jeffries *et al.*, 2015.

**B**

**A**


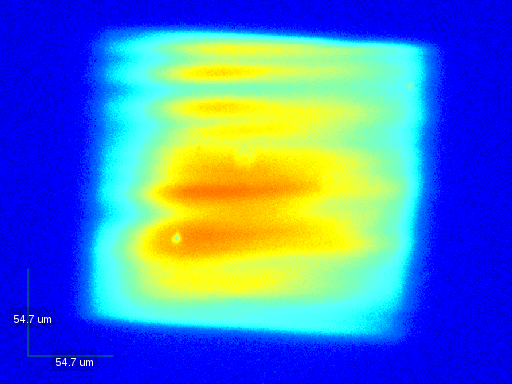

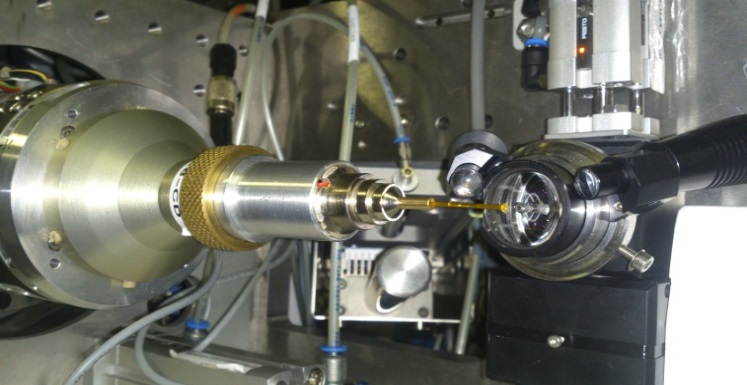


**Supplementary Figure 5.** Homogeneous irradiation of hAR solution for enzymatic experiments. (a) Homogeneous irradiation of the hAR solution was achieved using a top-hat square-shaped beam (200×180 μm^2^) as measured by a Ce:YAG fluorescence screen at the sample position, and scanning it through the axial section of the capillary. (b) The enzyme sample was inserted in a polyimide capillary and mounted in a SPINE magnetic base compatible with the goniometer available in the BL13-XALOC beamline at the ALBA synchrotron.


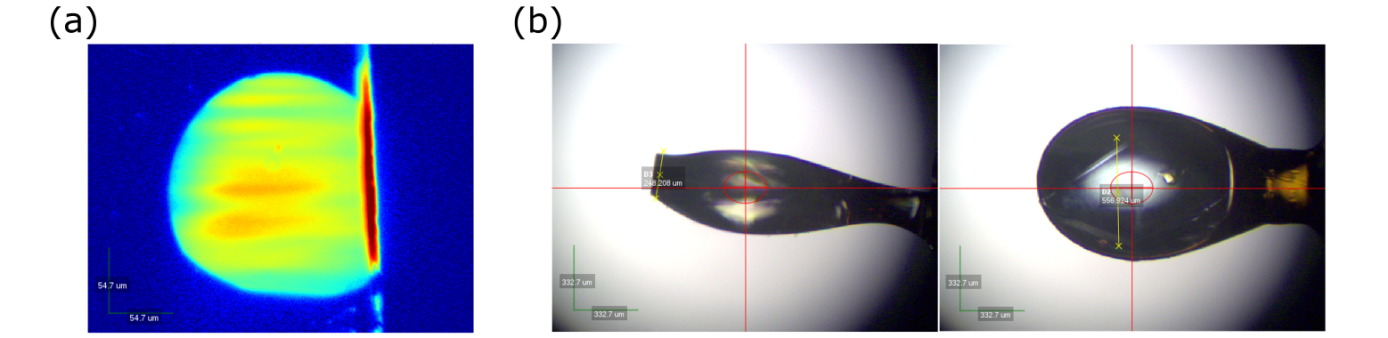


**B**

**A**

**Supplementary Figure 6.** Homogeneous irradiation of the crystal in MX experiments. (a) The crystal was exposed to a top-hat, circular-shaped X-ray beam in the BL13-XALOC beamline at the ALBA Synchrotron. The beam was circular with 190 µm in diameter, as measured by a Ce:YAG fluorescence screen at the sample position. The circular beam spot appears to be cut in the right side due to the rim of the fluorescence screen used exclusively to measure the beam size. (b) The hAR crystal as aligned with the X-ray beam.







**A**

**B**

**Supplementary Figure 7.** Calculated transmission coefficients. (a) Calculated transmission coefficient for the polyimide capillary (µ = 1.494 cm^2^/g and ρ = 1.425 g/cm^3^; R_int_ = 0.5 mm and R_out_ = 0.025 mm) used in the enzymatic experiments. (b) In pink: calculated transmission coefficient for a quartz capillary (µ = 9.804 cm^2^/g and ρ = 2.648 g/cm^3^; R_int_ = 0.8 mm and R_ext_ = 0.9 mm) used in SAXS experiments. In blue: calculated transmission coefficient for the sample (considered to be water: µ = 2.673 cm^2^/g and ρ = 1 g/cm^3^) used in SAXS experiments.


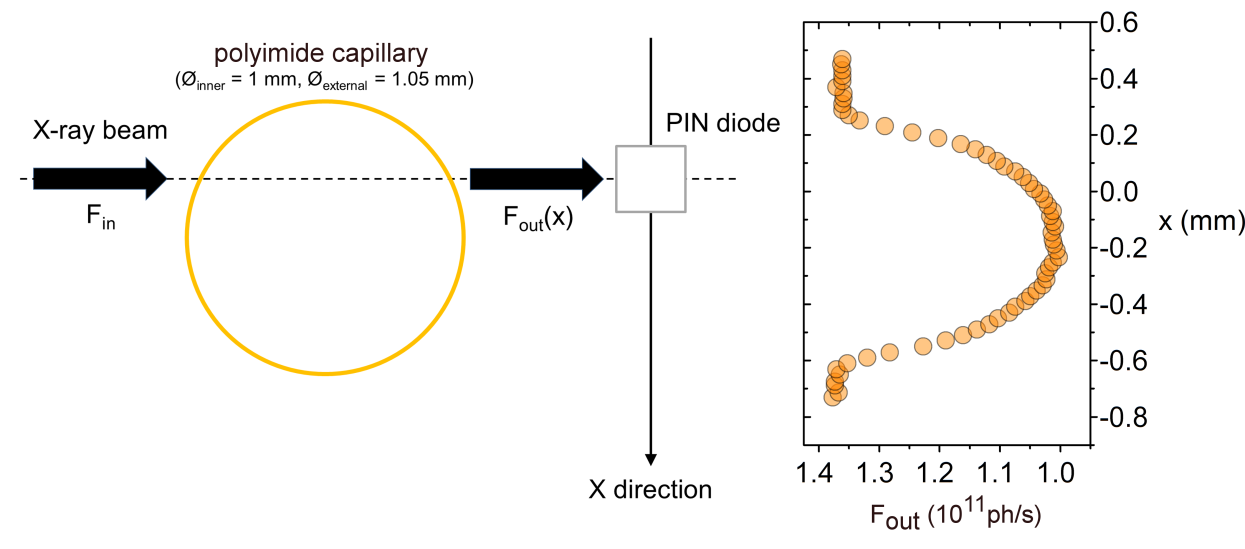


**Supplementary Figure 8.** Experimentally measured absorption in irradiation experiments of enzyme samples in solution. The control sample was inserted in a polyimide capillary and irradiated using a top-hat circular-shaped beam (Supplementary Fig. 6). The beam was scanned vertically and the transmitted flux was measured using a PIN diode placed after the capillary. The value extracted experimentally is compatible with the calculated transmissions.

| **MX data collection statistics** | | | | | | | | | | |
| --- | --- | --- | --- | --- | --- | --- | --- | --- | --- | --- |
| Dataset  Dose (MGy) | **D1**  0.03 | **D2**  0.21 | **D3**  0.39 | **D4**  0.57 | **D5**  0.75 | **D6**  0.93 | **D7**  1.11 | **D8**  1.29 | **D9**  1.47 | **D10**  1.65 |
|  |  |  |  |  |  |  |  |  |  |  |
| Resolution (Å) | 49.37-0.92 (0.94-0.92) | 49.38-0.94 (0.96-0.94) | 49.39-0.96 (0.98-0.96) | 49.41-0.96 (0.98-0.96) | 49.42-0.97 (0.99-0.97) | 49.43-0.98 (1.00-0.98) | 49.44-0.99 (1.01-0.99) | 49.45-1.01 (1.03-1.01) | 49.46-1.02 (1.04-1.02) | 49.47-1.03 (1.05-1.03) |
| R*_meas_* | 0.038 (0.352) | 0.037 (0.325) | 0.038 (0.306) | 0.039 (0.347) | 0.039 (0.340) | 0.040 (0.341) | 0.041 (0.342) | 0.041 (0.331) | 0.042 (0.341) | 0.043 (0.343) |
| Total number of observations | 754031 (33632) | 710512 (34066) | 667577 (29233) | 668072 (29091) | 650322 (30432) | 632865 (30825) | 614656 (30107) | 579058 (27350) | 563270 (25865) | 547873 (25354) |
| <I/σ_I_> | 18.9 (3.6) | 19.3 (4.0) | 19.5 (4.1) | 18.7 (3.7) | 18.3 (3.9) | 18.0 (3.9) | 17.6 (3.9) | 17.6 (4.1) | 17.2 (3.9) | 16.9 (4.0) |
| CC_1/2_ | 0.999 (0.889) | 0.999 (0.909) | 0.999 (0.916) | 0.999 (0.895) | 0.999 (0.895) | 0.999 (0.896) | 0.999 (0.900) | 0.999 (0.896) | 0.999 (0.891) | 0.999 (0.886) |
| Completeness (%) | 99.5 (96.7) | 99.6 (99.7) | 99.5 (95.8) | 99.5 (95.5) | 99.7 (99.8) | 99.7 (99.7) | 99.7 (99.7) | 99.7 (99.6) | 99.7 (99.6) | 99.7 (99.6) |
| Multiplicity | 3.6 (3.3) | 3.6 (3.5) | 3.6 (3.3) | 3.6 (3.3) | 3.6 (3.4) | 3.6 (3.6) | 3.6 (3.6) | 3.6 (3.5) | 3.6 (3.4) | 3.6 (3.4) |
| Mosaicity | 0.12 | 0.12 | 0.12 | 0.12 | 0.13 | 0.13 | 0.13 | 0.13 | 0.13 | 0.13 |
| Unit cell  a (Å)  b (Å)  c (Å)  β (º) | 47.35 66.60 49.40  92.05 | 47.36 66.62 49.41  92.05 | 47.36 66.63 49.42  92.03 | 47.37 66.64 49.44  92.03 | 47.37 66.65 49.45  92.02 | 47.37 66.66 49.46  92.02 | 47.37 66.67 49.47  92.02 | 47.37 66.68 49.48  92.02 | 47.38  66.69  49.49  92.01 | 47.38 66.69 49.50  92.01 |

| Dataset  Dose (MGy) | **D11**  1.83 | **D12**  2.01 | **D13**  2.19 | **D14**  2.37 | **D15**  2.55 | **D16**  2.73 | **D17**  2.91 | **D18**  3.09 | **D19**  3.27 | **D20**  3.45 |
| --- | --- | --- | --- | --- | --- | --- | --- | --- | --- | --- |
|  |  |  |  |  |  |  |  |  |  |  |
| Maximum resolution (Å) | 49.48-1.04 (1.06-1.04) | 49.49-1.06 (1.08-1.06) | 49.50-1.07 (1.09-1.07) | 49.50-1.08 (1.10-1.08) | 49.51-1.09 (1.11-1.09) | 49.52-1.11 (1.13-1.11) | 49.53-1.12 (1.14-1.12) | 49.54-1.13 (1.15-1.13) | 49.55-1.15 (1.17-1.15) | 49.55-1.17 (1.19-1.17) |
| *R_meas_* | 0.045 (0.347) | 0.045 (0.317) | 0.047 (0.322) | 0.047 (0.327) | 0.048 (0.341) | 0.048 (0.327) | 0.049 (0.334) | 0.049 (0.312) | 0.051 (0.333) | 0.051 (0.335) |
| Total number of observations | 532877 (24366) | 504817 (24543) | 489531 (21636) | 476693 (20243) | 464295 (19609) | 442939 (21812) | 431245 (21087) | 416503 (18088) | 398521 (19234) | 379104 (18037) |
| <I/σ_I_> | 16.5 (3.9) | 16.6 (4.5) | 16.3 (4.3) | 15.9 (4.2) | 15.6 (4.1) | 15.7 (4.7) | 15.4 (4.6) | 15.4 (4.7) | 15.1 (4.7) | 15.2 (4.6) |
| CC_1/2_ | 0.999 (0.889) | 0.999 (0.898) | 0.999 (0.900) | 0.999 (0.902) | 0.999 (0.892) | 0.999 (0.899) | 0.999 (0.898) | 0.999 (0.909) | 0.998 (0.905) | 0.999 (0.905) |
| Completeness (%) | 99.5 (95.9) | 99.6 (99.5) | 99.5 (95.8) | 99.5 (95.7) | 99.4 (94.9) | 99.6 (99.4) | 99.6 (99.5) | 99.2 (91.3) | 99.6 (99.3) | 99.6 (99.5) |
| Multiplicity | 3.6 (3.5) | 3.6 (3.6) | 3.6 (3.4) | 3.6 (3.3) | 3.6 (3.3) | 3.7 (3.6) | 3.7 (3.6) | 3.7 (3.5) | 3.7 (3.6) | 3.7 (3.5) |
| Mosaicity | 0.13 | 0.13 | 0.14 | 0.14 | 0.14 | 0.14 | 0.14 | 0.14 | 0.14 | 0.14 |
| Unit cell  a (Å)  b (Å)  c (Å)  β (º) | 47.38 66.70 49.51  92.01 | 47.38 66.71 49.51  92.01 | 47.38 66.71 49.52  92.01 | 47.38 66.72 49.53  92.01 | 47.38 66.73 49.54  92.00 | 47.38 66.74 49.55  92.00 | 47.38 66.74 49.55  92.00 | 47.38 66.75 49.56  92.00 | 47.38 66.76 49.57  92.00 | 47.38 66.77 49.58  92.00 |

**Supplementary Table 1.** Data collection statistics for the MX experiments. Macromolecular crystallography data collection statistics for all datasets D1-D20. Spatial group for all datasets is P2_1_; values in parenthesis refer to the highest resolution shell; $R_{meas}= \sum_{hkl} \left\{ N(hkl)/[N\left( hkl \right)-1] \right\}^{1/2}\times\sum_{i} \left| I_{i}\left( hkl \right)-\left\langle I\left( hkl \right) \right\rangle\right|/\sum_{hkl} \sum_{i} I_{i}(hkl)$.

| **Refinement statistics** | | | | | |
| --- | --- | --- | --- | --- | --- |
| **Dataset** | **D1** | **D5** | **D10** | **D15** | **D20** |
| Dose (MGy) | 0.03 | 0.75 | 1.65 | 2.55 | 3.45 |
| PDB code | 6F7R | 6F81 | 6F82 | 6F84 | 6F8O |
| Resolution range (Å) | 10 - 0.92 | 10 - 0.97 | 10 - 1.03 | 10 - 1.09 | 10 - 1.17 |
| No. reflections all/free | 177397/9549 | 153232/8104 | 127646/6716 | 107020/5563 | 86605/4591 |
| R*_work_*, R*_free_* | 0.104/0.131 | 0.108/0.135 | 0.110/0.139 | 0.120/0.152 | 0.119/0.163 |
| Average B factor, all atoms (Å^2^) | 13.0 | 14.0 | 15.0 | 16.0 | 19.0 |
| R.m.s. deviations | | |  |  |  |
| Bond lengths (Å) | 0.0158 | 0.0165 | 0.0164 | 0.0265 | 0.0178 |
| Bond angles (°) | 2.22 | 2.28 | 2.33 | 2.41 | 2.38 |
| Ramachandran Plot | | |  |  |  |
| Favoured (%) | 97 | 98 | 98 | 98 | 99 |
| Allowed (%) | 3 | 2 | 2 | 2 | 1 |
| Outliers (%) | 0 | 0 | 0 | 0 | 0 |

**Supplementary Table 2.** Refinement statistics for the MX experiments. Refinement statistics for datasets D1, D5, D10, D15 and D20. A random set of 5% of the reflections is selected to calculate$R_{free}= \sum_{hkl} \left| |F_{obs}(khl)|-|F_{calc}(khl)| \right|/\sum_{hkl} |F_{obs}(khl)|$; $R_{work}$ is calculated as for $R_{free}$ considering the reflections not selected in the test set; the number of reflections considered was selected following the $F_{obs}>4\sigma(F_{obs})$ cutoff.

|  | |  | RMSD | | |  |  |  |
| --- | --- | --- | --- | --- | --- | --- | --- | --- |
| D20 (with C298) *vs.* | |  | From Ser2 to Phe311 |  | Cys298 |  |  |  |
|  | |  |  |  |  |  |  |  |
| Zenarestat  (PDB 1IEI) | C_α_ atoms |  | 0.695 |  | 0.225 |  |  |  |
|  | Main chain |  | 0.722 |  | 1.718 |  |  |  |
|  | Side chain |  | 1.529 |  | 0.427 |  |  |  |
|  | All |  | 1.189 |  | 1.067 |  |  |  |
| Tolrestat  (PDB 1AH3) | C_α_ atoms |  | 0.558 |  | 0.767 |  |  |  |
|  | Main chain |  | 0.579 |  | 0.704 |  |  |  |
|  | Side chain |  | 1.142 |  | 0.915 |  |  |  |
|  | All |  | 0.893 |  | 0.808 |  |  |  |
| Epalrestat  (PDB 4JIR) | C_α_ atoms |  | 0.459 |  | 0.079 |  |  |  |
|  | Main chain |  | 0.470 |  | 0.103 |  |  |  |
|  | Side chain |  | 0.871 |  | 0.182 |  |  |  |
|  | All |  | 0.698 |  | 0.117 |  |  |  |
| FJ0048  (PDB 4XZH) | C_α_ atoms |  | 0.633 |  | 0.420 |  |  |  |
|  | Main chain |  | 0.639 |  | 0.384 |  |  |  |
|  | Side chain |  | 1.529 |  | 0.427 |  |  |  |
|  | All |  | 0.989 |  | 0.751 |  |  |  |
|  |  |  |  |  |  |  |  |  |

Supplementary Table 3. Alignment of the D20 dataset crystallographic structure *versus* the structures containing the various inhibitors. Root mean square deviations (RMSD), calculated for C_α_ atoms, main chains, side chains and all atoms, between the D20 dataset crystallographic structure and the structures with Zenarestat, Tolrestat, Epalrestat and JF0048 inhibitors. The structures were aligned using the whole structure excluding the disordered terminal residues (Ser2 to Phe311).

# References

[1] L. Misuri, M. Cappiello, F. Balestri, R. Moschini, V. Barracco, U. Mura, and A. Del-Corso, *J Enzyme Inhib Med Chem.***32**(1), 1152–1158 (2017).

1. Albert Castellví and Isidro Crespo contributed equally. [↑](#footnote-ref-1)
2. Present address: Deutsches Elektronen-Synchrotron DESY, Photon Science, Notkestrasse 85, 22607 Hamburg, Germany [↑](#footnote-ref-2)
